# Effectiveness of Extended Intravenous Antibiotic Prophylaxis in Preventing Periprosthetic Joint Infection After Total Knee and Hip Arthroplasty: An Analysis of a Japanese Nationwide Database

**DOI:** 10.2106/JBJS.OA.26.00212

**Published:** 2026-08-27

**Authors:** Shingo Kurihara, Chikamasa Ichita, Tadahiro Goto, Kazuhisa Hatayama, Kiyohide Fushimi, Sayuri Shimizu

**Affiliations:** 1 Department of Health Data Science, Graduate School of Data Science, Yokohama City University, Yokohama, Japan; 2 Department of Orthopaedic Surgery, Hokusuikai Kinen Hospital, Mito, Japan; 3 Department of Gastroenterology, Kitasato University School of Medicine, Sagamihara, Japan; 4 Department of Orthopaedic Surgery, Japan Community Health Care Organization Gunma Central Hospital, Maebashi, Japan; 5 Department of Health Policy and Informatics, Graduate School of Medicine, Institute of Science Tokyo, Tokyo, Japan

## Abstract

**Background:**

Although major international guidelines recommend discontinuing antibiotic prophylaxis within 24 hours after total joint arthroplasty, recent studies have suggested potential benefits of extended administration. The aim of this study was to evaluate the effectiveness of extended intravenous antibiotic prophylaxis in preventing periprosthetic joint infection (PJI).

**Methods:**

The Japanese Diagnosis Procedure Combination database was used to identify adults who underwent primary total knee arthroplasty (TKA) and total hip arthroplasty (THA) from April 2014 to March 2024. Based on the duration of postoperative intravenous antibiotic prophylaxis, patients were divided into the standard intravenous antibiotic prophylaxis (0-1 day) and extended intravenous antibiotic prophylaxis (2-3 days) groups. Patients who received any oral antibiotic prophylaxis during the intravenous prophylactic period were excluded. The outcome was PJI within 90 and 365 days after surgery, defined as surgical site infection requiring surgical intervention. Propensity score matching and subgroup analyses in high-risk patients were conducted (e.g., obesity, smoking, diabetes mellitus with chronic complications, kidney disease, hepatic disease, autoimmune disease, and oral corticosteroid use).

**Results:**

Among 506,710 eligible patients (244,156 TKA and 262,554 THA), the 90-day PJI incidence was 0.34% in TKA and 0.37% in THA. In propensity score–matched analysis, extended intravenous antibiotic prophylaxis was associated with a small reduction in 90-day PJI after TKA, with a risk difference (RD) of −0.07% (95% confidence interval [CI], −0.12% to −0.01%) and a corresponding risk ratio (RR) of 0.83 (95% CI, 0.72-0.96). In high-risk TKA patients, a stronger association was observed (RD, −0.13% [95% CI, −0.25% to −0.01%]; RR, 0.78 [95% CI, 0.63-0.98]). Similar findings were observed for 365-day PJI after TKA. No clear association was observed in THA, either in the overall cohort or in the high-risk subgroup.

**Conclusions:**

Extended intravenous antibiotic prophylaxis was associated with a marginal reduction in PJI within 90 and 365 days after TKA, with a greater effect in high-risk TKA patients. While routine extension is not supported for all patients, it may be considered in selected high-risk TKA cases.

**Level of Evidence:**

Therapeutic Level Ⅲ. See Instructions for Authors for a complete description of levels of evidence.

## Introduction

Periprosthetic joint infection (PJI) is an uncommon but serious complication following total joint arthroplasty (TJA), with incidence ranging from 0.26% to 2.4%^1-4^. PJI is associated with multiple reoperations^5,6^, poor functional recovery and satisfaction^7-9^, prolonged hospital stays, and increased healthcare costs^10-12^, highlighting the importance of preventing PJI. Among standardized infection-prevention strategies widely implemented in TJA, perioperative antibiotic prophylaxis is a key element. Major international guidelines recommend starting intravenous antibiotic prophylaxis just before surgery and discontinuing within 24 hours postoperatively^13-16^. At the same time, they recommend against the extension of antibiotics beyond 24 hours due to lack of evidence for additional benefit in preventing surgical site infections^17,18^. Concerns also include antimicrobial resistance^19-21^, antibiotic-related adverse effects^18,22,23^, and increased healthcare costs^24,25^.

Despite these recommendations, 2 key questions remain unresolved: the effectiveness of extending intravenous antibiotic prophylaxis beyond 24 hours and its benefit in high-risk patients. The evidence supporting discontinuation within 24 hours is derived from diverse surgical procedures including non-TJA surgeries and principles of antimicrobial stewardship, rather than TJA-specific data^13-16^. While recent systematic reviews and meta-analyses have suggested a potential benefit of extended prophylaxis^1,26^, these studies included heterogeneous protocols combining intravenous and oral antibiotics. This heterogeneity obscures the isolated effectiveness of extending intravenous prophylaxis duration, which is the core component of perioperative antimicrobial prophylaxis. In addition, although some studies suggest a potential preventive effect in high-risk patients^1,27^, the specific efficacy in these patients remains unclear due to insufficient risk stratification. Given the low incidence of PJI, large-scale real-world data with clear definition of intravenous prophylaxis duration and stratification of high-risk populations are needed to address these gaps.

The purpose of this study was to evaluate whether extended intravenous antibiotic prophylaxis was associated with a lower incidence of PJI following TJA compared with discontinuation within 24 hours. Using a large-scale nationwide database, we examined outcomes in both the overall population and high-risk subgroups to address gaps in the current evidence.

## Materials and Methods

### Design and Setting, Data Source

We conducted a retrospective cohort study using the Japanese Diagnosis Procedure Combination (DPC) database, a national administrative claims database covering >1,000 acute care hospitals^28^. Validation studies reported >96% specificity and 50% to 80% sensitivity for major diagnoses, with surgical procedure codes achieving >90% sensitivity and specificity^29^.

This database contains comprehensive patient summaries and detailed medical service utilization records, with diagnoses coded using the International Classification of Diseases, 10th Revision (ICD-10). After discharge, patient data are only available when readmitted to the same hospital. This study was approved by the Institutional Review Board of Yokohama City University (F250300049) with a waiver of informed consent because the data were anonymized. This study was reported in accordance with the Strengthening the Reporting of Observational Studies in Epidemiology (STROBE) and REporting of studies Conducted using Observational Routinely-collected health Data (RECORD) guidelines.

### Participants

Between April 1, 2014, and March 31, 2024, we identified all patients who underwent primary total knee arthroplasty (TKA) and total hip arthroplasty (THA) (Supplemental Table I). The following patients were excluded: (1) data outliers (height <130 or >200 cm; weight <30 or >200 kg), (2) age <18 years, (3) staged bilateral TJA (performed on different days) during the same hospitalization, (4) with nonstandard prophylactic antibiotics (standard agents were defined as first- or second-generation cephalosporins, penicillins, glycopeptides, lincomycin derivatives, and fosfomycin)^2,15^, (5) no intravenous prophylaxis on the day of surgery, (6) continuous intravenous prophylaxis extended beyond postoperative day 4, and (7) oral antibiotics administered during the intravenous prophylaxis period or the following day. To assess the isolated effect of intravenous prophylaxis duration, we excluded patients who received oral antibiotics on postoperative days 0 to 2 when intravenous prophylaxis lasted 0 to 1 day or on postoperative days 0 to 4 when intravenous prophylaxis lasted 2 to 3 days.

### Collected Variables

Patient characteristics included age, sex, body mass index (BMI) (kg/m^2^), smoking history (nonsmoker, past/current smoker, missing data), Barthel index^30^, academic hospital status, and preexisting comorbidities: hypertension, heart disease, respiratory disease, hepatic disease, renal disease, diabetes mellitus with complications, and autoimmune disease^31^ (Supplemental Table I). The Charlson Comorbidity Index score was categorized into 3 groups (≤1, 2, and ≥3)^32,33^. As perioperative factors, corticosteroid injection on the day of surgery, oral corticosteroid prescription during hospitalization, bilateral surgery, cell saver use, autologous transfusion, and cement use were counted. In the DPC database, we could not distinguish between antibiotic-loaded and plain cement as these were the same claim code. The annual number of TKA or THA procedures performed at each hospital was categorized into quartiles (very low, low, medium, and high)^4,34,35^. The year of surgery was extracted to account for temporal trends.

Based on previous reports^36-41^, we defined high-risk patients as those with at least one of the following conditions: obesity (BMI > 35), diabetes mellitus with chronic complications, smoking, kidney disease, hepatic disease, autoimmune disease, or oral corticosteroid intake. Since controlled diabetes mellitus alone may not significantly increase infection risk, only diabetes mellitus with chronic complications was included^42-44^.

### Exposure

Intravenous antibiotic prophylaxis was defined as continuous administration from the day of surgery, while administration with a gap of 1 or more days was considered therapeutic and excluded from this analysis. Patients were divided into 2 groups according to intravenous antibiotic prophylaxis duration: standard prophylaxis (postoperative days 0-1) and extended prophylaxis (postoperative days 2-3). The 0- to 1-day cutoff aligns with major international guidelines recommending discontinuation within 24 hours^13-16^. The 2- to 3-day cutoff reflects the Japanese clinical practice guideline, which recommends prophylaxis for up to 48 hours for TJA^45^, and a previous study evaluated intravenous antibiotic prophylaxis duration using a cutoff at postoperative day 3^46^.

### Outcomes

The primary outcome was PJI within 90 days after surgery^3,4^. Secondary outcomes included PJI during the index hospitalization and within 365 days after surgery, as well as antibiotic-related complications. PJI was defined as a diagnosis of surgical site infection (SSI) identified using ICD-10 codes in combination with any surgical intervention, including wound debridement, arthroscopic or open synovectomy, removal of implants, or revision arthroplasty (Supplemental Table I)^4,47^. Surgical procedures recorded in the DPC database have been previously validated^48^. This definition does not capture cases managed with antibiotic suppression alone without surgical intervention.

Patients with early postoperative infection signs may have received extended antibiotics therapeutically rather than prophylactically. To address this reverse causation and immortal-time bias, we performed a landmark analysis at postoperative day 4, excluding cases reoperated on days 0 to 3.

For assessment of antibiotic-related complications, we counted *Clostridioides difficile* colitis, acute kidney injury, acute liver injury, and severe allergic reactions, identified using ICD-10 codes (Supplemental Table I).

### Statistical Analysis

First, in the unmatched cohort, we summarized baseline characteristics and reoperation date distributions stratified by prophylaxis duration. Continuous variables are presented as means with standard deviations and categorical variables as numbers with percentages. Baseline differences between groups were evaluated using standardized mean differences (SMD). Missing Barthel index values were imputed using median imputation (proportion of missing: 0.80%).

Second, propensity scores were estimated using logistic regression, with all collected variables described above as covariates (see Collected Variables subsection). One-to-one matching between the 0- to 1-day and 2- to 3-day groups was performed using the nearest neighbor method without replacement, with a caliper width of 0.2 of the standard deviation of the estimated propensity scores. Covariate balance was assessed using SMD, with < 0.1 considered adequate. After propensity score matching, we compared standard (0-1 day) vs. extended (2-3 days) intravenous antibiotic prophylaxis. Risk ratios (RRs) and risk differences (RDs) with 95% confidence intervals (CIs) were estimated using generalized estimating equations to account for residual hospital-level clustering^49^. For the estimation of RRs, Poisson regression models with a log link function were used, whereas RDs were estimated using models with an identity link function. An independence working correlation structure was specified, with robust sandwich variance estimators applied to obtain valid standard errors. All analyses were two-sided with significance set at p < 0.05.

We also conducted subgroup analyses limited to patients at high risk of infection. Within the high-risk subgroup, propensity score–matched analyses were performed, with propensity scores re-estimated specifically for the high-risk population. Analyses were performed using R software version 4.5.2.

## Results

### Study Flow

**Fig. 1:**
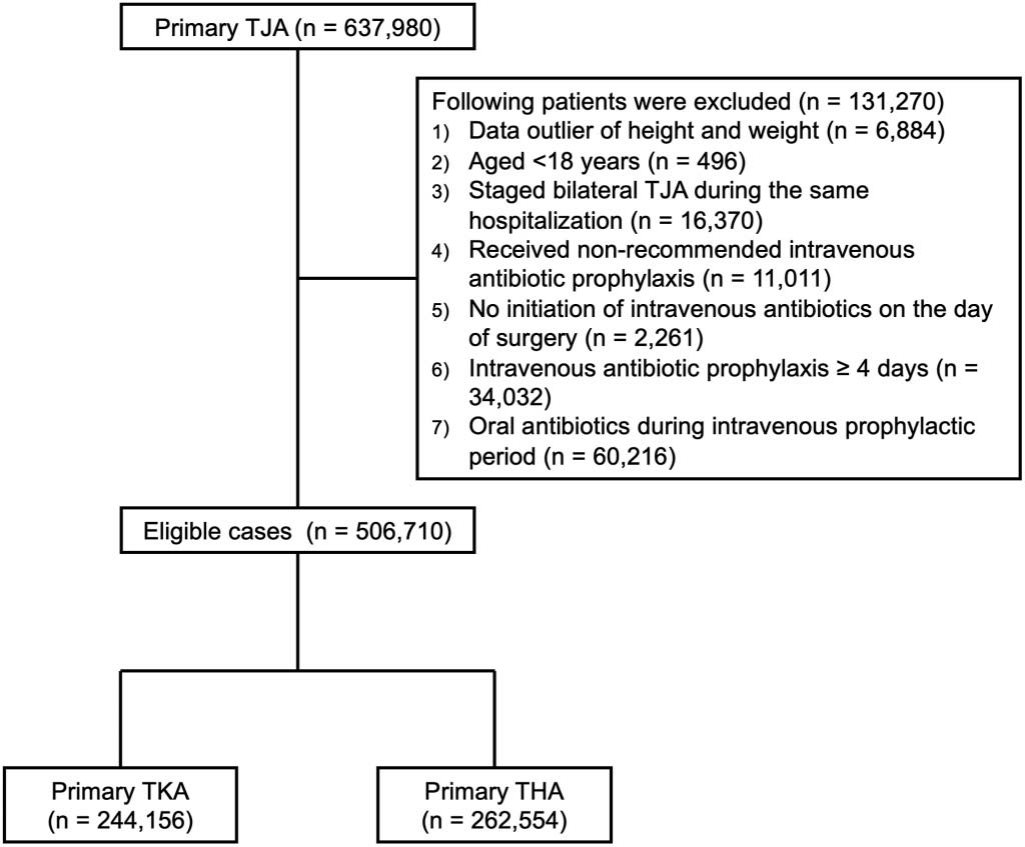
Flowchart of patients. THA, total hip arthroplasty, TJA = total joint arthroplasty, and TKA = total knee arthroplasty.

Among 637,980 cases, 131,270 were excluded, and the remaining 506,710 eligible cases included 244,156 TKA and 262,554 THA procedures (Fig. 1).

### Baseline Characteristics

In TKA, the mean age was 74.8 years, 79.1% were women, and the median length of stay was 24 days; 55.9% received standard prophylaxis, and 44.1% received extended prophylaxis. In THA, the mean age was 67.9 years, 81.4% were women, and the median length of stay was 22 days; 62.4% received standard prophylaxis, and 37.6% received extended prophylaxis. In both TKA and THA, the proportion of standard prophylaxis increased over the study period, whereas the use of extended prophylaxis decreased.

Propensity score matching yielded 103,790 TKA pairs and 98,527 THA pairs, with all SMDs below 0.1 except for the year of surgery in TKA (SMD = 0.123) and autologous transfusion in THA (SMD = 0.196) (Table I).

**Table I:** Baseline Characteristics Before and After Propensity Score Matching

Variables	Total Knee Arthroplasty						Total Hip Arthroplasty					
	Unmatched			Matched			Unmatched			Matched		
	Standard ≤1 day (n = 136,457)	Extended 2–3 days (n = 107,699)	SMD	Standard ≤1 day (n = 103,790)	Extended 2–3 days (n = 103,790)	SMD	Standard ≤1 day (n = 163,766)	Extended 2–3 days (n = 98,788)	SMD	Standard ≤1 day (n = 98,527)	Extended 2–3 days (n = 98,527)	SMD
Age (years), mean (SD)	74.7 (7.8)	74.9 (7.7)	0.027	74.9 (7.8)	74.9 (7.7)	0.009	67.9 (10.9)	67.9 (11.0)	<0.001	68.0 (11.0)	67.9 (11.0)	0.008
Women, n (%)	107,992 (79.1)	85,110 (79.0)	0.003	81,884 (78.9)	81,550 (78.6)	<0.001	133,624 (81.6)	80,122 (81.1)	0.013	79,737 (80.9)	79,906 (81.1)	0.004
Body mass index, mean (SD)	26.2 (4.3)	26.1 (4.2)	0.016	26.1 (4.2)	26.1 (4.2)	0.003	24.0 (4.2)	24.0 (4.2)	0.001	24.0 (4.2)	24.0 (4.2)	0.003
Smoking history, n (%)			0.042			0.018			0.049			0.025
Nonsmoker	108,256 (79.3)	87,187 (81.0)		83,148 (80.1)	83,879 (80.8)		121,080 (73.9)	75,056 (76.0)		74,960 (76.1)	74,836 (76.0)	
Past/current smoker	20,020 (14.7)	14,745 (13.7)		14,759 (14.2)	14,187 (13.7)		31,182 (19.0)	17,621 (17.8)		18,005 (18.3)	17,580 (17.8)	
Missing data	8,181 (6.0)	5,767 (5.4)		5,883 (5.7)	5,724 (5.5)		11,504 (7.0)	6,111 (6.2)		5,562 (5.6)	6,111 (6.2)	
Charlson Comorbidity Index, n (%)			0.010			0.016			0.017			0.005
≤1	76,778 (56.3)	60,994 (56.6)		58,715 (56.6)	59,431 (57.3)		111,724 (68.2)	66,650 (67.5)		66,222 (67.2)	66,444 (67.4)	
2	39,717 (29.1)	31,299 (29.1)		30,124 (29.0)	29,422 (28.3)		35,167 (21.5)	21,567 (21.8)		21,698 (22.0)	21,528 (21.8)	
≥3	19,962 (14.6)	15,406 (14.3)		14,951 (14.4)	14,937 (14.4)		16,875 (10.3)	10,571 (10.7)		10,607 (10.8)	10,555 (10.7)	
Barthel index, mean (SD)	95.9 (10.7)	95.8 (10.6)	0.005	95.9 (10.5)	95.8 (10.6)	0.004	95.7 (11.8)	95.2 (12.1)	0.041	95.5 (11.8)	95.2 (12.1)	0.020
Comorbid conditions, n (%)												
Hypertension	51,471 (37.7)	39,147 (36.3)	0.028	38,973 (37.5)	37,656 (36.3)	0.026	40,076 (24.5)	23,943 (24.2)	0.005	25,064 (25.4)	23,906 (24.3)	0.027
Heart disease	5,023 (3.7)	3,861 (3.6)	0.005	3,768 (3.6)	3,759 (3.6)	<0.001	4,815 (2.9)	3,142 (3.2)	0.014	3,022 (3.1)	3,139 (3.2)	0.007
Respiratory disease	23,144 (17.0)	18,572 (17.2)	0.008	17,689 (17.0)	17,825 (17.2)	0.003	18,338 (11.2)	11,692 (11.8)	0.020	11,538 (11.7)	11,678 (11.9)	0.004
Hepatic disease	3,504 (2.6)	2,730 (2.5)	0.002	2,607 (2.5)	2,601 (2.5)	<0.001	3,853 (2.4)	2,521 (2.6)	0.013	2,504 (2.5)	2,520 (2.6)	0.001
Kidney disease	3,670 (2.7)	2,411 (2.2)	0.029	2,406 (2.3)	2,374 (2.3)	0.002	3,483 (2.1)	1,905 (1.9)	0.014	1,932 (2.0)	1,905 (1.9)	0.002
Diabetes with chronic complications	1,898 (1.4)	1,615 (1.5)	0.009	1,495 (1.4)	1,576 (1.5)	0.006	1,354 (0.8)	803 (0.8)	0.002	793 (0.8)	799 (0.8)	0.001
Autoimmune disease	11,069 (8.1)	8,149 (7.6)	0.020	8,668 (8.4)	7,800 (7.5)	0.031	8,366 (5.1)	5,276 (5.3)	0.010	5,523 (5.6)	5,265 (5.3)	0.012
Oral corticosteroid intake, n (%)	4,429 (3.2)	3,497 (3.2)	<0.001	3,268 (3.1)	3,412 (3.3)	0.008	5,898 (3.6)	4,056 (4.1)	0.026	3,897 (4.0)	4,055 (4.1)	0.008
Perioperative procedures, n (%)												
Corticosteroid injection	60,989 (44.7)	40,367 (37.5)	0.147	41,734 (40.2)	38,946 (37.5)	0.055	46,833 (28.6)	24,752 (25.1)	0.080	24,781 (25.2)	24,733 (25.1)	0.001
Same-day bilateral surgery	10,604 (7.8)	6,209 (5.8)	0.080	5,434 (5.2)	6,181 (6.0)	0.031	5,866 (3.6)	2,306 (2.3)	0.074	2,033 (2.1)	2,306 (2.3)	0.019
Cell saver use	13,658 (10.0)	7,019 (6.5)	0.127	9,126 (8.8)	6,754 (6.5)	0.086	38,293 (23.4)	21,202 (21.5)	0.046	19,658 (20.0)	21,156 (21.5)	0.038
Autologous transfusion	28,370 (20.8)	23,990 (22.3)	0.036	22,521 (21.7)	23,155 (22.3)	0.015	68,487 (41.8)	51,555 (52.2)	0.209	41,851 (42.5)	51,424 (52.2)	0.196
Cement use	122,968 (90.1)	96,865 (89.9)	0.006	94,645 (91.2)	93,083 (89.7)	0.051	33,971 (20.7)	15,210 (15.4)	0.139	15,294 (15.5)	15,210 (15.4)	0.002
Facility conditions, n (%)												
Academic hospital	16,926 (12.4)	18,539 (17.2)	0.136	16,035 (15.4)	17,473 (16.8)	0.038	34,347 (21.0)	25,413 (25.7)	0.112	23,759 (24.1)	25,152 (25.5)	0.033
Annual hospital volume of surgery*			0.296			0.054			0.240			0.042
Very low	28,222 (20.7)	29,833 (27.7)		27,649 (26.6)	28,131 (27.1)		35,503 (21.7)	28,225 (28.6)		27,945 (28.4)	28,178 (28.6)	
Low	31,367 (23.0)	32,962 (30.6)		30,023 (28.9)	31,282 (30.1)		37,515 (22.9)	27,319 (27.7)		28,882 (29.3)	27,105 (27.5)	
Medium	38,075 (27.9)	21,788 (20.2)		23,700 (22.8)	21,417 (20.6)		44,472 (27.2)	22,491 (22.8)		21,437 (21.8)	22,491 (22.8)	
High	38,793 (28.4)	23,116 (21.5)		22,418 (21.6)	22,960 (22.1)		46,276 (28.3)	20,753 (21.0)		20,263 (20.6)	20,753 (21.1)	
Surgery year, n (%)			0.271			0.123			0.254			0.068
2014	8,274 (6.1)	11,705 (10.9)		8,196 (7.9)	11,549 (11.1)		9,290 (5.7)	9,007 (9.1)		7,650 (7.8)	8,977 (9.1)	
2015	10,371 (7.6)	11,196 (10.4)		10,004 (9.6)	10,638 (10.2)		11,369 (6.9)	9,821 (9.9)		8,788 (8.9)	9,699 (9.8)	
2016	11,808 (8.7)	11,557 (10.7)		10,593 (10.2)	10,481 (10.1)		13,310 (8.1)	10,301 (10.4)		9,979 (10.1)	10,233 (10.4)	
2017	12,762 (9.4)	11,326 (10.5)		10,633 (10.2)	10,130 (9.8)		14,908 (9.1)	10,731 (10.9)		10,634 (10.8)	10,690 (10.8)	
2018	13,982 (10.2)	12,057 (11.2)		10,910 (10.5)	11,457 (11.0)		16,347 (10.0)	11,159 (11.3)		11,104 (11.3)	11,159 (11.3)	
2019	14,027 (10.3)	9,867 (9.2)		9,905 (9.5)	9,682 (9.3)		16,598 (10.1)	8,449 (8.6)		9,065 (9.2)	8,449 (8.6)	
2020	15,429 (11.3)	10,096 (9.4)		10,735 (10.3)	10,053 (9.7)		18,134 (11.1)	9,053 (9.2)		9,525 (9.7)	9,053 (9.2)	
2021	15,397 (11.3)	9,758 (9.1)		10,775 (10.4)	9,746 (9.4)		19,815 (12.1)	9,392 (9.5)		10,022 (10.2)	9,392 (9.5)	
2022	17,134 (12.6)	10,317 (9.6)		11,254 (10.8)	10,277 (9.9)		21,287 (13.0)	10,480 (10.6)		10,898 (11.1)	10,480 (10.6)	
2023	17,273 (12.7)	9,820 (9.1)		10,785 (10.4)	9,777 (9.4)		22,708 (13.9)	10,395 (10.5)		10,862 (11.0)	10,395 (10.6)	

Patients who received additional oral antibiotic prophylaxis were excluded from each cohort.

TKA: very low (1-27), low (28-53), medium (54-97), high (98-360). THA: very low (1-34), low (35-74), medium (76-139), high (140-527).

SMD = standardized mean difference, THA = total hip arthroplasty, and TKA = total knee arthroplasty.

* Annual hospital volume of surgery was categorized separately for each procedure.

### Outcomes

**Fig. 2:**
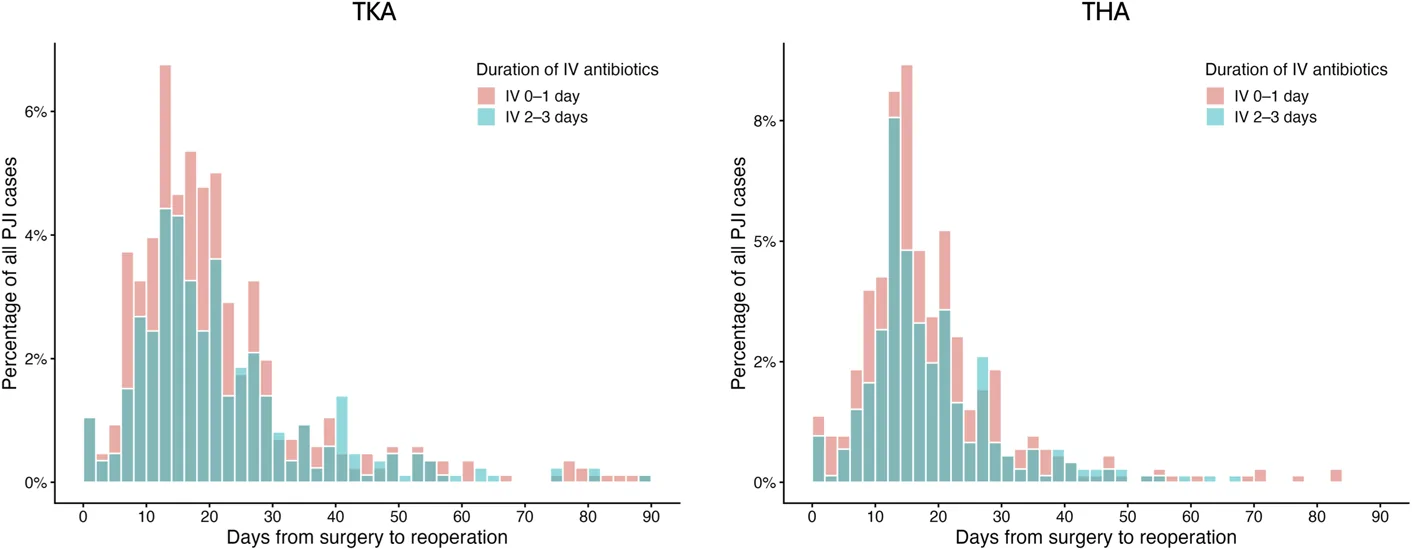
Distribution of reoperation date after surgery stratified by the duration of intravenous antibiotic prophylaxis in the overall unmatched cohort. IV = intravenous, PJI = periprosthetic joint infection, THA = total hip arthroplasty, and TKA = total knee arthroplasty.

In the overall cohort, the 90-day PJI incidence was 0.34% in TKA and 0.37% in THA. PJI-related reoperations on postoperative days 0 to 3 accounted for 4.2% of all PJI in TKA and 3.7% in THA (TKA: 13 vs. 12 cases; THA: 14 vs. 8 cases in standard vs. extended groups). The distribution of postoperative days to reoperation in the unmatched cohort is shown in Figure 2. Reoperation peaked around 10 to 20 days after surgery, with no apparent difference in distribution between groups.

After propensity score matching, extended prophylaxis was associated with a lower 90-day PJI incidence in TKA, although the absolute RD was small (RD, −0.07%; 95% CI, −0.12% to −0.01%; RR, 0.83; 95% CI, 0.72-0.96). By contrast, among THA patients, extended prophylaxis was not significantly associated with 90-day PJI (RD, −0.01%; 95% CI, −0.06% to 0.04%; RR, 0.97; 95% CI, 0.84-1.12). Findings at 365 days were consistent with the 90-day analysis in both TKA (RR, 0.81; 95% CI, 0.71-0.93) and THA (RR, 1.02; 95% CI, 0.90-1.16) (Table II). Extended prophylaxis did not increase antibiotic-related complications in both TKA and THA (Supplemental Table II).

**Table II:** Propensity Score–Matched Outcomes Comparing Intravenous Antibiotic Duration in the Overall Cohort

Outcomes, n (%)	Total Knee Arthroplasty					Total Hip Arthroplasty				
	Standard ≤1 day (n = 103,790)	Extended 2 to 3 days (n = 103,790)	Risk Ratio (95% CI)	Risk Difference (95% CI)	p	Standard ≤1 day (n = 98,527)	Extended 2 to 3 days (n = 98,527)	Risk ratio (95% CI)	Risk Difference (95% CI)	p
PJI in index hospitalization	374 (0.36)	311 (0.30)	0.83 (0.72 to 0.97)	−0.06 (−0.11 to −0.01)	0.02	268 (0.27)	287 (0.29)	1.07 (0.91 to 1.26)	0.02 (−0.03 to 0.07)	0.42
PJI within 90 days	397 (0.39)	329 (0.32)	0.83 (0.72 to 0.96)	−0.07 (−0.12 to −0.01)	0.01	384 (0.39)	374 (0.38)	0.97 (0.84 to 1.12)	−0.01 (−0.06 to 0.04)	0.72
PJI within 365 days	449 (0.44)	364 (0.36)	0.81 (0.71 to 0.93)	−0.08 (−0.14 to −0.03)	<0.01	474 (0.48)	484 (0.49)	1.02 (0.90 to 1.16)	0.01 (−0.05 to 0.07)	0.75

CI = confidence interval, and PJI = periprosthetic joint infection.

### Subgroup Analysis

High-risk patients accounted for 28.7% of both TKA and THA. In TKA, the mean age was 72.5 years with 63.4% women, whereas in THA, the mean age was 65.0 years with 64.3% women. The most common risk factors were smoking (TKA 14.2%, THA 18.6%), autoimmune disease (TKA 7.9%, THA 5.2%), and obesity (TKA 3.3%, THA 1.6%). The 90-day PJI incidences were 0.52% in TKA and 0.60% in THA.

Propensity score matching yielded 29,798 TKA pairs and 27,922 THA pairs, with all SMDs below 0.1 except for surgery year in TKA (SMD = 0.111) and autologous transfusion in THA (SMD = 0.187) (Supplemental Table III). Extended intravenous prophylaxis was associated with a lower 90-day PJI rate in TKA (RD, −0.13%; 95% CI, −0.25% to −0.01%; RR, 0.78; 95% CI, 0.63-0.98), but not in THA (RD, −0.05%; 95% CI, −0.17% to 0.08%; RR, 0.93; 95% CI, 0.75-1.15). Findings at day 365 were similar to the 90-day analysis in both TKA (RR, 0.81; 95% CI, 0.65-0.99) and THA (RR, 0.99; 95% CI, 0.81-1.19) (Table III).

**Table III:** Propensity Score–Matched Outcomes Comparing Intravenous Antibiotic Duration in the High-Risk Subgroup

Outcomes, n (%)	Total Knee Arthroplasty					Total Hip Arthroplasty				
	Standard ≤1 day (n = 29,798)	Extended 2 to 3 days (n = 29,798)	Risk ratio (95% CI)	Risk difference (95% CI)	p	Standard ≤1 day (n = 27,922)	Extended 2 to 3 days (n = 27,922)	Risk ratio (95% CI)	Risk difference (95% CI)	p
PJI in index hospitalization	165 (0.56)	132 (0.45)	0.80 (0.64 to 1.01)	−0.11 (−0.23 to 0.00)	0.06	120 (0.43)	125 (0.45)	1.04 (0.81 to 1.34)	0.02 (−0.09 to 0.13)	0.75
PJI within 90 days	179 (0.61)	140 (0.48)	0.78 (0.63 to 0.98)	−0.13 (−0.25 to −0.01)	0.03	174 (0.62)	161 (0.58)	0.93 (0.75 to 1.15)	−0.05 (−0.17 to 0.08)	0.48
PJI within 365 days	195 (0.66)	157 (0.54)	0.81 (0.65 to 0.99)	−0.13 (−0.25 to −0.00)	0.04	212 (0.76)	209 (0.75)	0.99 (0.81 to 1.19)	−0.01 (−0.15 to 0.13)	0.88

CI = confidence interval, and PJI = periprosthetic joint infection.

## Discussion

In this study, we examined the association between postoperative intravenous antibiotic duration and PJI using a large-scale data set. Extended intravenous antibiotic prophylaxis (2-3 days) was associated with a statistically significant but marginal reduction in PJI incidence in TKA, whereas no significant association was observed in THA. Furthermore, this association was stronger in high-risk TKA patients, and no significant increase in antibiotic-related complications was observed.

The findings of this study are largely consistent with existing international guidelines that recommend against prolonged antibiotic prophylaxis beyond 24 hours after TJA^13-16^. However, our results highlight that the infection-preventive effect of extended intravenous prophylaxis is not uniform but varies by surgical procedure and patient risk status. In the overall TKA population, the absolute RD associated with extended intravenous antibiotic administration was 0.07%, corresponding to a number needed to treat (NNT) of 1,428. Although statistically significant, this marginal reduction does not support routine extension of intravenous prophylaxis. By contrast, among high-risk patients, the reduction was 0.13% with an NNT of 769. This estimate is comparable with estimates reported in prior economic evaluations of extended oral antibiotic prophylaxis in high-risk populations, which demonstrated cost-effectiveness at an absolute risk reduction of 0.187% for TKA^50^. While derived from different settings, these findings suggest that extended prophylaxis may warrant consideration in selected high-risk patients. Further refinement of risk stratification is needed to identify subgroups most likely to benefit.

This study has several limitations. First, despite propensity score matching, selection bias and unmeasured confounders related to the retrospective design cannot be entirely excluded. Second, the possibility of reverse causation, which means prolonged antibiotic use due to early infection, cannot be completely ruled out. To address this, we performed landmark analysis at day 4 and excluded cases reoperated on days 0 to 3. Third, PJI incidence may have been underestimated because our outcome definition required both SSI diagnosis and reoperation, potentially missing cases managed without surgical intervention. Fourth, because our PJI definition included wound debridement, some superficial SSI cases may have been misclassified as PJI. Fifth, the identification of antibiotic-related complications relied solely on ICD-10 codes, which lack robust validation in the DPC database. Sixth, the DPC database has a single code for bone cement, preventing distinction between antibiotic-loaded and plain cement, so residual confounding cannot be excluded. Seventh, to isolate the effect of intravenous antibiotic prophylaxis, patients who received oral antibiotics were excluded. As a result, the study population may differ slightly from the overall TJA population, which may limit generalizability. Finally, the DPC database lacked race or ethnicity data, limiting generalizability of our findings to other racial and ethnic groups.

## Conclusions

In this large-scale nationwide study, extended intravenous antibiotic prophylaxis was associated with a small reduction in PJI within 90 and 365 days after TKA, with a greater effect in high-risk patients, whereas no clear benefit was observed in THA. Given the minimal absolute risk reduction in the overall population, routine extension of intravenous antibiotics beyond 24 hours is not supported for all patients. However, extended prophylaxis may be considered in selected high-risk TKA cases.

## Appendix

Supporting material provided by the authors is posted with the online version of this article as a data supplement at jbjs.org (https://links.lww.com/JBJSOA/B311). This content was not copyedited or verified by JBJS.

## References

[1] Dasari SP Kanumuri SD Yang J Manner PA Fernando ND Hernandez NM. Extended prophylactic antibiotics for primary and aseptic revision total joint arthroplasty: a meta-analysis. J Arthroplasty. 2024;39(9):S476-87. 38237874 10.1016/j.arth.2024.01.014

[2] Siddiqi A Forte SA Docter S Bryant D Sheth NP Chen AF. Perioperative antibiotic prophylaxis in total joint arthroplasty: a systematic review and meta-analysis. J Bone Joint Surg. 2019;101(9):828-42. 31045673 10.2106/JBJS.18.00990

[3] Heckmann ND Wang JC Piple AS Marshall GA Mills ES Liu KC Lieberman JR Christ AB. Is intraoperative dexamethasone utilization associated with increased rates of periprosthetic joint infection following total joint arthroplasty? J Arthroplasty. 2023;38(2):224-31.e1. 36031084 10.1016/j.arth.2022.08.028

[4] Kurihara S Ichita C Goto T Hatayama K Fushimi K Shimizu S. Association between intraoperative periarticular injection of triamcinolone and early postoperative infection in total knee arthroplasty: an analysis of a Japanese nationwide database. J Arthroplasty. 2025;40(10):2615-22.e3. 40280208 10.1016/j.arth.2025.04.041

[5] Nelson SB Pinkney JA Chen AF Tande AJ. Periprosthetic joint infection: current clinical challenges. Clin Infect Dis. 2023;77(7):e34-45. 37434369 10.1093/cid/ciad360 PMC11004930

[6] Fink B Schlumberger M. Antibiotic therapy alone does not have a high success rate in cases of unexpected positive cultures in intraoperative samples from hip and knee prosthesis revision. BMC Musculoskelet Disord. 2020;21(1):786. 33248455 10.1186/s12891-020-03799-w PMC7700714

[7] Kuiper JWP Rustenburg CME Willems JH Verberne SJ Peters EJG Saouti R. Results and patient reported outcome measures (PROMs) after one-stage revision for periprosthetic joint infection of the hip: a single-centre retrospective study. J Bone Joint Infect. 2018;3(3):143-9. 10.7150/jbji.24366 PMC6043468 30013896

[8] Wildeman P Rolfson O Söderquist B Wretenberg P Lindgren V. What are the long-term outcomes of mortality, quality of life, and hip function after prosthetic joint infection of the hip? A 10-year follow-up from Sweden. Clin Orthop Relat Res. 2021;479(10):2203-13. 34061486 10.1097/CORR.0000000000001838 PMC8445574

[9] Manning L Rofe A Athan E Gill SD Yates P Cooper D Davis JS Aboltins C. Patient-reported outcomes following periprosthetic joint infection of the hip and knee: a longitudinal, prospective observational study. J Bone Joint Surg. 2024;106(13):1197-204. 38723046 10.2106/JBJS.23.00717

[10] Morcos MW Kooner P Marsh J Howard J Lanting B Vasarhelyi E. The economic impact of periprosthetic infection in total knee arthroplasty. Can J Surg. 2021;64(2):E144-8. 33666386 10.1503/cjs.012519 PMC8064239

[11] Alp E Cevahir F Ersoy S Guney A. Incidence and economic burden of prosthetic joint infections in a university hospital: a report from a middle-income country. J Infect Public Health. 2016;9(4):494-8. 26829894 10.1016/j.jiph.2015.12.014

[12] Kim HS Park JW Moon SY Lee YK Ha YC Koo KH. Current and future burden of periprosthetic joint infection from national claim database. J Korean Med Sci. 2020;35(49):e410. 33350183 10.3346/jkms.2020.35.e410 PMC7752258

[13] Berríos-Torres SI Umscheid CA Bratzler DW Leas B Stone EC Kelz RR Reinke CE Morgan S Solomkin JS Mazuski JE Dellinger EP Itani KMF Berbari EF Segreti J Parvizi J Blanchard J Allen G Kluytmans JAJW Donlan R Schecter WP; Healthcare Infection Control Practices Advisory Committee. Centers for disease control and prevention guideline for the prevention of surgical site infection, 2017. JAMA Surg. 2017;152(8):784-91. 28467526 10.1001/jamasurg.2017.0904

[14] Allegranzi B Bischoff P de Jonge S Kubilay NZ Zayed B Gomes SM Abbas M Atema JJ Gans S van Rijen M Boermeester MA Egger M Kluytmans J Pittet D Solomkin JS; WHO Guidelines Development Group. New WHO recommendations on preoperative measures for surgical site infection prevention: an evidence-based global perspective. Lancet Infect Dis. 2016;16(12):e276-87. 27816413 10.1016/S1473-3099(16)30398-X

[15] Bratzler DW Dellinger EP Olsen KM Perl TM Auwaerter PG Bolon MK Fish DN Napolitano LM Sawyer RG Slain D Steinberg JP Weinstein RA. Clinical practice guidelines for antimicrobial prophylaxis in surgery. Am J Health Syst Pharm. 2013;70(3):195-283. 23327981 10.2146/ajhp120568

[16] Prokuski L. Prophylactic antibiotics in orthopaedic surgery. J Am Acad Orthop Surg. 2008;16(5):283-93. 18460689 10.5435/00124635-200805000-00007

[17] De Jonge SW Boldingh QJJ Solomkin JS Dellinger EP Egger M Salanti G Allegranzi B Boermeester MA. Effect of postoperative continuation of antibiotic prophylaxis on the incidence of surgical site infection: a systematic review and meta-analysis. Lancet Infect Dis. 2020;20(10):1182-92. 32470329 10.1016/S1473-3099(20)30084-0

[18] Mirahmadi A Hosseini-Monfared P Farrokhi M Minaie R Bateni A Kazemi SM. Extended oral antibiotics fail to reduce surgical site infection in orthopedic surgeries: a comparative study. PLoS One. 2025;20(9):e0330685. 40911548 10.1371/journal.pone.0330685 PMC12412922

[19] Harbarth S Samore MH Lichtenberg D Carmeli Y. Prolonged antibiotic prophylaxis after cardiovascular surgery and its effect on surgical site infections and antimicrobial resistance. Circulation. 2000;101(25):2916-21. 10869263 10.1161/01.cir.101.25.2916

[20] Scuderi GR Vatti L Mont MA Parvizi J. Extended oral antibiotic prophylaxis in total joint arthroplasty may be challenging antimicrobial stewardship. J Arthroplasty. 2025;40(5):1109-11. 40023459 10.1016/j.arth.2025.02.072

[21] Llor C Bjerrum L. Antimicrobial resistance: risk associated with antibiotic overuse and initiatives to reduce the problem. Ther Adv Drug Saf. 2014;5(6):229-41. 25436105 10.1177/2042098614554919 PMC4232501

[22] Oppelaar MC Zijtveld C Kuipers S Ten Oever J Honings J Weijs W Wertheim HFL. Evaluation of prolonged vs short courses of antibiotic prophylaxis following ear, nose, throat, and oral and maxillofacial surgery: a systematic review and meta-analysis. JAMA Otolaryngol Head Neck Surg. 2019;145(7):610. 31070697 10.1001/jamaoto.2019.0879 PMC6512286

[23] Branch-Elliman W O'Brien W Strymish J Itani K Wyatt C Gupta K. Association of duration and type of surgical prophylaxis with antimicrobial-associated adverse events. JAMA Surg. 2019;154(7):590. 31017647 10.1001/jamasurg.2019.0569 PMC6487902

[24] Bin Akresh A Alzeet DS Alshalan NZ Alhawsawi MM Alsaleh NA Binsuwaidan RA Alhubaishi AA Altowaijri AI Alebedah NN Sherbeeni NM Alnajjar LI. The impact of antibiotic prophylaxis appropriateness, duration and associated cost on the rate of surgical site infection at a tertiary hospital in Riyadh, Saudi Arabia. Saudi Med J. 2025;46(6):688-94. 40516930 10.15537/smj.2025.46.6.20241061 PMC12199636

[25] Clean Cut Investigators Group. An observational cohort study on the effects of extended postoperative antibiotic prophylaxis on surgical-site infections in low- and middle-income countries. Br J Surg. 2024;111(1):znad438. 38198157 10.1093/bjs/znad438 PMC10782210

[26] Yakkanti RR Vanden Berge D Summers SH Mansour KL Lavin AC Hernandez VH. Extended postoperative prophylactic antibiotics for primary and aseptic revision total joint arthroplasty: a systematic review. J Am Acad Orthop Surg. 2022;30(11):e822-32. 35245256 10.5435/JAAOS-D-21-00977

[27] Villa JM Pannu TS Braaksma W Higuera CA Riesgo AM. Extended oral antibiotic prophylaxis after aseptic total hip or knee arthroplasty revisions: a preliminary report. J Arthroplasty. 2023;38(1):141-5. 35952854 10.1016/j.arth.2022.08.003

[28] Yasunaga H. Updated information on the diagnosis procedure combination data. Ann Clin Epidemiol. 2024;6(4):106-10. 39726797 10.37737/ace.24015 PMC11668689

[29] Yamana H Moriwaki M Horiguchi H Kodan M Fushimi K Yasunaga H. Validity of diagnoses, procedures, and laboratory data in Japanese administrative data. J Epidemiol. 2017;27(10):476-82. 28142051 10.1016/j.je.2016.09.009 PMC5602797

[30] Shah S Vanclay F Cooper B. Improving the sensitivity of the Barthel Index for stroke rehabilitation. J Clin Epidemiol. 1989;42(8):703-9. 2760661 10.1016/0895-4356(89)90065-6

[31] Tunnicliffe L Warren-Gash C. Clinical Codelist- Autoimmune Disease ICD10 Codes. London, UK: London School of Hygiene & Tropical Medicine; 2022.

[32] Honda A Iizuka Y Michihata N Uda K Mieda T Takasawa E Ishiwata S Kakuta Y Tomomatsu Y Ito S Inomata K Matsui H Fushimi K Yasunaga H Chikuda H. Effect of intraoperative tranexamic acid on perioperative major hemorrhage requiring transfusion in patients undergoing elective spine surgery: a propensity score-matched analysis using a national inpatient database. Glob Spine J. 2024;14(3):804-11. 10.1177/21925682221123317 PMC11192125 36006871

[33] Quan H Li B Couris CM Fushimi K Graham P Hider P Januel JM Sundararajan V. Updating and validating the Charlson comorbidity index and score for risk adjustment in hospital discharge abstracts using data from 6 countries. Am J Epidemiol. 2011;173(6):676-82. 21330339 10.1093/aje/kwq433

[34] Brodeur PG Kim KW Modest JM Cohen EM Gil JA Cruz AI. Surgeon and facility volume are associated with postoperative complications after total knee arthroplasty. Arthroplasty Today. 2022;14:223-30.e1. 35510066 10.1016/j.artd.2021.11.017 PMC9059075

[35] Laucis NC Chowdhury M Dasgupta A Bhattacharyya T. Trend toward high-volume hospitals and the influence on complications in knee and hip arthroplasty. J Bone Joint Surg. 2016;98(9):707-12. 27147682 10.2106/JBJS.15.00399 PMC4850659

[36] Inabathula A Dilley JE Ziemba-Davis M Warth LC Azzam KA Ireland PH Meneghini RM. Extended oral antibiotic prophylaxis in high-risk patients substantially reduces primary total hip and knee arthroplasty 90-day infection rate. J Bone Joint Surg. 2018;100(24):2103-9. 30562290 10.2106/JBJS.17.01485

[37] Moon YW Kim YS Kwon SY Kim SY Lim SJ Park YS. Perioperative risk of hip arthroplasty in patients with cirrhotic liver disease. J Korean Med Sci. 2007;22(2):223-6. 17449928 10.3346/jkms.2007.22.2.223 PMC2693586

[38] Kunutsor SK Whitehouse MR Blom AW Beswick AD; INFORM Team. Patient-related risk factors for periprosthetic joint infection after total joint arthroplasty: a systematic review and meta-analysis. PLoS One. 2016;11(3):e0150866. 26938768 10.1371/journal.pone.0150866 PMC4777569

[39] Quan H Sundararajan V Halfon P Fong A Burnand B Luthi JC Saunders LD Beck CA Feasby TE Ghali WA. Coding algorithms for defining comorbidities in ICD-9-CM and ICD-10 administrative data. Med Care. 2005;43(11):1130-9. 16224307 10.1097/01.mlr.0000182534.19832.83

[40] Kheir MM Dilley JE Ziemba-Davis M Meneghini RM. The AAHKS clinical research award: extended oral antibiotics prevent periprosthetic joint infection in high-risk cases: 3855 patients with 1-year follow-up. J Arthroplasty. 2021;36(7):S18-25. 33589279 10.1016/j.arth.2021.01.051 PMC9161732

[41] Shih LY Cheng CY Chang CH Hsu KY Hsu RWW Shih HN. Total knee arthroplasty in patients with liver cirrhosis. J Bone Joint Surg. 2004;86(2):335-41. 14960679 10.2106/00004623-200402000-00017

[42] Pulido L Ghanem E Joshi A Purtill JJ Parvizi J. Periprosthetic joint infection: the incidence, timing, and predisposing factors. Clin Orthop Relat Res. 2008;466(7):1710-5. 18421542 10.1007/s11999-008-0209-4 PMC2505241

[43] Adams AL Paxton EW Wang JQ Johnson ES Bayliss EA Ferrara A Nakasato C Bini SA Namba RS. Surgical outcomes of total knee replacement according to diabetes status and glycemic control, 2001 to 2009. J Bone Joint Surg. 2013;95(6):481-7. 23446446 10.2106/JBJS.L.00109 PMC6948790

[44] Marchant MH Viens NA Cook C Vail TP Bolognesi MP. The impact of glycemic control and diabetes mellitus on perioperative outcomes after total joint arthroplasty. J Bone Joint Surg Am. 2009;91(7):1621-9. 19571084 10.2106/JBJS.H.00116

[45] Japanese Society of Chemotherapy; Japanese Society for Surgical Infection. Practical Guidelines for the Appropriate Use of Antimicrobial Prophylaxis in Surgery. Tokyo: Japanese Society of Chemotherapy; 2016 [in Japanese]. Available at: https://www.chemotherapy.or.jp/uploads/files/guideline/jyutsugo_shiyou_jissen.pdf. Accessed April 26, 2026.

[46] Shang J Wang L Gong J Su D Jia X Wang Y. Impact of antibiotic prophylaxis courses on postoperative complications following total joint arthroplasty: finding from Chinese population. J Clin Pharm Ther. 2022;47(1):61-9. 34664290 10.1111/jcpt.13545

[47] Yamagami R Inui H Jo T Kawata M Taketomi S Kono K Kawaguchi K Sameshima S Kage T Matsui H Fushimi K Yasunaga H Tanaka S. Unicompartmental knee arthroplasty is associated with lower proportions of surgical site infection compared with total knee arthroplasty: a retrospective nationwide database study. Knee. 2021;28:124-30. 33359944 10.1016/j.knee.2020.11.017

[48] Konishi T Yoshimoto T Fujiogi M Yamana H Tanabe M Seto Y Yasunaga H. Validity of operative information in Japanese administrative data: a chart review-based analysis of 1221 cases at a single institution. Surg Today. 2022;52(10):1484-90. 35552817 10.1007/s00595-022-02521-8

[49] Hubbard AE Ahern J Fleischer NL der Laan MV Lippman SA Jewell N Bruckner T Satariano WA. To GEE or not to GEE: comparing population average and mixed models for estimating the associations between neighborhood risk factors and health. Epidemiology. 2010;21(4):467-74. 20220526 10.1097/EDE.0b013e3181caeb90

[50] Lipson S Pagani NR Moverman MA Puzzitiello RN Menendez ME Smith EL. The cost-effectiveness of extended oral antibiotic prophylaxis for infection prevention after total joint arthroplasty in high-risk patients. J Arthroplasty. 2022;37(10):1961-6. 35472436 10.1016/j.arth.2022.04.025

